# The S/S Genotype of the 5-HTTLPR (Serotonin-Transporter-Linked Promoter Region) Variant of the SLC6A4 Gene Decreases the Risk of Pre-Eclampsia

**DOI:** 10.3390/jpm13111535

**Published:** 2023-10-26

**Authors:** Rebeca Mónica Ramírez-Armas, Idalia Garza-Veloz, Juan Carlos Olivas-Chávez, Rosa Martha Covarrubias-Carrillo, Maria Calixta Martínez-Vázquez, Joel Monárrez-Espino, Anayantzin E. Ayala-Haro, Claudia Vanessa Serrano-Amaya, Ivan Delgado-Enciso, Iram Pablo Rodriguez-Sanchez, Margarita L. Martinez-Fierro

**Affiliations:** 1 Molecular Medicine Laboratory, Academic Unit of Human and Health Sciences, Autonomous University of Zacatecas, Zacatecas 98160, Mexico; rebecam.ra7@gmail.com (R.M.R.-A.); idaliagv@uaz.edu.mx (I.G.-V.); rosmarth.covarru@uaz.edu.mx (R.M.C.-C.); calibgst@uaz.edu.mx (M.C.M.-V.); jmonarrez@hotmail.com (J.M.-E.); anayantzinayala@gmail.com (A.E.A.-H.); serranoamaya93@gmail.com (C.V.S.-A.); 2 Hospital de la Mujer Zacatecana, Servicios de Salud de Zacatecas, Zacatecas 98600, Mexico; olijco@yahoo.com.mx; 3 Department of Health Research, Christus Muguerza del Parque Hospital, Chihuahua 31000, Mexico; 4 School of Medicine, University of Colima, Colima 28040, Mexico; ivan_delgado_enciso@ucol.mx; 5 Cancerology State Institute, Colima State Health Services, Colima 28085, Mexico; 6 Laboratorio de Fisiología Molecular y Estructural, Facultad de Ciencias Biológicas, Universidad Autónoma de Nuevo León, San Nicolás de los Garza 66455, Mexico; iram.rodriguezsa@uanl.edu.mx

**Keywords:** pre-eclampsia, serotonin, 5-HTTLPR, SLC6A4 gene

## Abstract

Pre-eclampsia (PE) is a disorder characterized by hypertension in the second trimester of pregnancy that results from abnormal placentation affecting fetal development and maternal health. Previous studies have shown the role of serotonin (5-HT) that leads to poor placental perfusion, where S/S and S/L polymorphisms promote the solute carrier family 6 member 4 (SLC6A4) gene associated with the risk of developing changes in the microvasculature of the placenta. This study looked at the association between the gene variant 5-HTTLPR (serotonin-transporter-linked promoter region) of the SLC6A4 gene and the occurrence of PE. A total of 200 women were included: 100 cases (pregnant with PE) and 100 controls (pregnant without complications). Genotyping of the 5-HTTLPR variant was performed using polymerase chain reaction (PCR). Associations between the presence of the genetic variant of interest and PE and other clinical features were evaluated statistically. The frequencies of S/S, S/L, and L/L genotypes were 32%, 53%, and 15% for the cases and 55%, 25%, and 20% in the control group. Compared to the controls, the genotype frequencies S/S vs. S/L + L/L (recessive model) in the cases group were different (*p* = 0.002). The S/S genotype decreased the probability of PE (OR = 0.39, 95% IC: 0.22–0.69, *p* = 0.002) and PE with severity criteria (OR = 0.39, 95% IC: 0.17–0.91, *p* = 0.045). The 5-HTTLPR gene variant of the SLC6A4 gene modifies the risk of PE development among the studied population.

## 1. Introduction

Pre-eclampsia (PE) is a pregnancy-specific disorder associated with new-onset hypertension, which occurs after 20 weeks of gestation (WG) [1]. Worldwide, PE is one of the leading causes of maternal and fetal morbidity and mortality [2,3,4]. Maternal mortality related to PE is estimated to be around 2–16.0% of all pregnancies [1].

Maternal clinical findings of PE include hypertension (≥140/90 mmHg), proteinuria (≥0.3 g/day), and/or other features such as thrombocytopenia, microangiopathic hemolysis, hepatic dysfunction, renal failure, pulmonary edema, headache, epigastric pain, and novo cerebral or visual disturbance [5,6]. In the fetus, PE can cause intrauterine growth restriction (IUGR), and it is also associated with decreased amniotic fluid, placental abruption, premature delivery, and hypoxemia [7,8]. So far, there is no effective treatment for PE; fetal delivery is the usual management. PE is associated with a later risk of hypertension in the early following years [9,10], increased risk of ischemic heart disease, stroke, and death from cardiovascular causes [11,12], a 5- to 12-fold increased risk of developing chronic kidney disease later in life [13,14], unfavorable metabolic profiles, with insulin resistance, elevated cholesterol and triglycerides, and elevated body mass index (BMI) [15]. In the offspring, PE is associated with later cardiovascular disease, neurological deficits [16,17,18,19,20,21], and hypertension [22,23,24]. As for the effects on offspring, there are cardiovascular effects, with an increase in blood pressure in offspring exposed to PE, that may be mediated, at least in part, by the effect of PE on IUGR [25]. The kidney may be programmed by various perinatal insults, such as placental insufficiency. In rodent models of placental insufficiency, a decrease in the number of nephrons and consequent alteration in blood pressure regulation in the offspring has always been found [26,27]. Moreover, it has been found that intrauterine exposure to PE had an adverse effect on the cognitive function of the offspring, as well as a statistically significant increase in the odds of autism spectrum disorder in PE-exposed offspring [25]. On the other hand, Tuovinen et al. also evaluated depressive symptoms in children exposed to PE, and they demonstrated that participants born after a primiparous pregnancy complicated by PE had over 30% higher depressive symptom scores in adulthood compared with those born after a primiparous normotensive pregnancy [28,29].

While there are multiple theories for the etiology of PE, the main cause relates to a deficient placentation process. In a normal pregnancy, trophoblastic invasion occurs in two stages: the first appears at 8–10 WG, transforming the spiral arteries of the decidua, and the second transforms the spiral arteries of the myometrium at 16–18 WG. In both stages, the cytotrophoblastic cells invade the arterial wall and increase their diameter, allowing adequate placental blood flow [30]. In PE, a failure during the second stage leads to an aberrant transformation of the spiral arteries, causing placental ischemia–hypoperfusion, oxidative stress, and endothelial dysfunction, with a release of soluble antiangiogenic factors into the circulation originating the clinical manifestations of the disease [30,31].

PE can have an early or late onset, range from mild to severe illness, and be associated with various maternal predisposing factors, including chronic hypertension, pre-existing diabetes mellitus, multifetal pregnancies, nutritional factors, maternal immunological alterations, genetic susceptibility, and history of PE in the previous pregnancy [32,33,34,35,36]. In terms of genetic susceptibility, serotonergic dysregulation appears to play a role in the etiology of PE [37,38,39].

Since the 1960s, serotonin (5-HT) was proposed as a factor involved in poor placental perfusion, as it was found in the placentas of women with PE [40]. Later, the study of the 5-HT transporter gene (5-HTT or SERT) variants showed a relationship between the risk of developing changes in the placental microvasculature [38,41] and the 5-HTT expression in the apical membrane of the placental syncytiotrophoblast [42,43]. Tryptophan, a precursor of 5-HT, also revealed its regulatory role in promoting better maternal–fetal tolerance [44].

5-HT transport is performed by the 5-HTT, which regulates intracellular (in neurons and platelets) and extracellular (in synaptic cleft and plasma) 5-HT concentrations, namely, 5-HT function [45]. Several genetic variants of the 5-HTT-linked promoter region (5-HTTLPR) have been identified [46]. Of these, the most studied is a guanine- and cytosine-rich region called 5-HTTLPR, located 1.2 kb upstream of the gene promoter, where relatively common allelic forms (528 bp “L” and 484 bp “S”) of a GC-rich repetitive sequence have been described [46]. Transfection studies in lymphoblastoid cells report that individuals carrying the L allele show increased transcriptional activity and basal uptake of 5-HT compared with cells carrying the S allele. Thus, this variant has the property of modulating the ability to reuptake 5-HT [46]. L/L is associated with a three-fold higher transcriptional activity and greater responsiveness to stimulation by forskolin or phorbol ester, which is significantly more potent in the suppression of transcriptional activity when fused to a heterozygous promoter [47]. Cells with L/L genotype also uptake more 5-HT compared with S/S or L/S cells [48]. In addition, the L variant is significantly more potent than the S variant in transcription in heterologous expression systems; greater availability of SERT has been found in the raphe of L/L cells compared with S/S cells [49]. Regarding transcription and 5-HT uptake, the S/S and L/S genotypes show similar data but differ from L/L, suggesting that the variant has a dominant-recessive effect [50,51]. According to the above, and considering the important role of 5-HT during normal pregnancy and the effect of the gene variants of the 5-HTT on the bio-availability of local and systemic 5-HT, the aim of this study was to evaluate the association between the gene variant 5-HTTLPR of the SLC6A4 gene and the occurrence of PE in a sample of the Mexican population.

## 2. Materials and Methods

### 2.1. Study Design and Participants

This was a case–control study. Patient information was extracted from the databases of the Molecular Medicine Laboratory of the Academic Unit of Human Medicine and Health Sciences of the Autonomous University of Zacatecas Francisco García Salinas, in Zacatecas, Mexico (AUHMHS-UAZ). The participants were drawn from a cohort of 588 pregnant women followed from the first trimester to delivery in the Jose Castro Villagrana Health Center and in the Hospital de la Mujer Zacatecana between November 2011 and January 2014 [52]. Cases (*n* = 100) were patients older than 18 years with a diagnosis of PE, with a single pregnancy, who agreed to participate and provided informed consent. PE was defined and classified according to the guidelines of the American College of Obstetricians and Gynecologists (ACOG) [1,53]. Controls (*n* = 100) were normotensive pregnant women with a single fetus and older than 18 years. The exclusion criteria for both groups were as follows: women with a history of previous abortions and any comorbidity diagnosed before or during the course of gestation, in vitro pregnancy, and multiple pregnancy. Patients whose DNA sample was insufficient to genotype the variant of interest and/or who presented pregnancy with a fetus with chromosomal or structural abnormalities were excluded. Biochemical parameters were obtained from the medical records of each patient.

### 2.2. Criteria for Pre-Eclampsia Classification

The following were the signs and symptoms described to classify patients with severity criteria according to the ACOG guidelines [1]: persistent headache or other cerebral or visual disturbances; epigastric or right upper quadrant pain; nausea and vomiting; pulmonary edema or cyanosis; hypertension ≥ 160/110 mmHg; proteinuria ± 5 g in 24 h urine or 3+ on dipstick in two random samples collected 4 h apart; oliguria (<500 mL/24 h); serum creatinine > 1.1 mg/dL; increase in either liver enzyme, alanine transaminase (ALT) and aspartate transaminase (AST), or both; thrombocytopenia < 100,000/mm^3^; micro-angiopathic hemolytic anemia, evidenced by increased lactate dehydrogenase (LHD) concentration; IUGR; oligohydramnios; absence of fetal movements; and fetal death.

Mild and severe PE was also considered according to the previous PE severity criteria [53]. Briefly, PE was considered severe if one or more of the following criteria was present: Blood pressure of 160 mm Hg systolic or higher or 110 mm Hg diastolic or higher on two occasions at least 6 h apart while the patient is on bed rest; proteinuria of 5 g or higher in a 24 h urine specimen or 3+ or greater on two random urine samples collected at least 4 h apart, oliguria of less than 500 mL in 24 h, cerebral or visual disturbances, pulmonary edema or cyanosis, epigastric or right upper quadrant pain, impaired liver function, thrombocytopenia, and/or IUGR [53]. Mild pre-eclampsia was considered when blood pressure did not exceed 160/110 mmHg and proteinuria was less than 5 g in a 24 h urine sample, taking into account mainly proteinuria [53]. This classification was updated by ACOG [1], taking into account other parameters of the symptomatology mentioned in the first part of this section. In this study, for the PE classification according to the time of onset, the cut-off point was 32 WG [53,54].

### 2.3. Biological Samples

Stored maternal DNA samples (−80 °C) from peripheral blood were obtained from the Bank of Biological Samples of the Molecular Medicine Laboratory of the AUHMHS-UAZ. Samples were provided by pregnant women as part of a screening study for adverse pregnancy outcomes in Zacatecas, Mexico. This study was conducted in accordance with the Declaration of Helsinki and was approved by the Institutional Research Ethics Committee (Protocol IDs: R-2010-1905-17; Ofc. Nos. 0072009, 0062010; HMZ520/281/11; and HMZ-5020/318/11).

Prior to the assessment of the genotypes of the interest variant, 100 additional DNA samples from the Molecular Medicine Laboratory bank were used to evaluate deviations from the Hardy–Weinberg (HW) law of the 5-HTTLPR genetic variant. These samples were obtained from healthy and unrelated Mexican donors from Zacatecas state. The HW equilibrium was evaluated by an exact test (see the Section 2.5 for details).

### 2.4. Genotyping of the 5-HTTLPR Variant of the SLC6A4 Gene by Polymerase Chain Reaction (PCR)

Frozen DNA samples were thawed at RT and mixed with vortex. An amount of 1.5 μL of DNA from each sample was quantified in a NanoDrop spectrophotometer (NanoDrop Technologies, Wilmington, DE, USA). The concentration and purity of each sample were obtained by means of its software. Genotyping of the 5-HTTLPR variant was carried out by end-point polymerase chain reaction (PCR) according to the protocol described by Heils et al. [55]. The following PCR primers were designed using the NCBI primer blast program: sense 5′-GGCGTTGCCGCTCTGAATGC-3′ and antisense 5′-GAGGGACTGAGCTGGACAACCAC-3′. The PCR conditions were carried out in 10 μL of the final volume, 1X Master Mix (Promega: Madison, WI, USA), 0.5 μM of each primer (T4 Oligo: Irapuato, Mexico), and 100 ng of template DNA. The thermocycling was carried out in the thermocycler (TECHNE Staffordshire, UK) with an initial cycle of at 95 °C for 3 min followed by 40 cycles, each with the following characteristics: 30 s of denaturation at 95 °C, 30 s of alignment at 60 °C, 35 s of extension at 72 °C and final step at 72 °C for 3 min was added.

**Figure 1:**
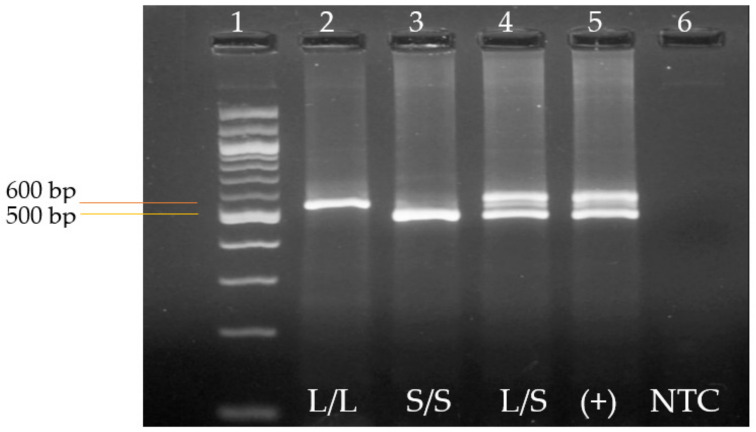
Electrophoresis of the 5-HT-transporter-linked promoter region (5-HTTLPR) gene variant. A 3% agarose gel is shown. Lane 1 shows 100 bp DNA molecular weight marker; lanes 2 and 3 show PCR products of patients with genotype homozygous L/L (528 bp) and S/S (484 bp); lane 4 shows genotype heterozygous S/L; and lanes 5 and 6 show positive control and non-template control, respectively.

PCR products were then subjected to 3% agarose gel electrophoresis (Cleaver Scientific, Rugby, UK) stained with ethidium bromide. Electrophoresis was run at 88 V for 1 h 30 min to achieve adequate band separation. The expected electrophoresis bands are 528 bp for L/L and 484 bp for S/S (Figure 1). A 100 bp molecular weight marker (Promega, Madison, WI, USA) was used to identify the amplicons corresponding to the L/L, S/L, and S/S genotypes. These results were observed with the UVP High-Performance UV Transilluminator, using the UVP framework.exe software (Figure 1).

### 2.5. Statistical Analysis

The patients’ general characteristics, biochemical data, and clinical information related to the disease were compared between study groups. For the analysis of nominal variables, frequency tables were built, and chi-squared and Fisher’s exact tests were used. For normally distributed continuous variables, Student *t*-tests to compare means were used, and non-parametric Mann–Whitney *U* tests were used for variables with skewed distribution. Odds ratios (ORs) with 95% confidence intervals (CIs) were computed, and Yates’ continuity correction was applied. The equilibrium of HW was evaluated by an exact test; this technique establishes that mattings occur at random and are not subject to mutation, selection, or migration so that genotypic frequencies remain constant from one generation to the next. It is said that a population is in equilibrium when the alleles of polymorphic systems maintain their frequency in the population through the generations [56]. For statistical analysis, the statistical package Sigma Plot version 14 was used, and *p* < 0.05 was considered significant.

## 3. Results

### 3.1. General and Clinical Characteristics of the Study Population

Table 1 shows the general characteristics of the study population. The mean maternal age was 28 and 25 years for cases and controls, respectively. A history of first-degree PE was present in 8% of cases and 9% of controls. It was the first pregnancy for nearly half of the cases (47%). The average gestational age at PE diagnosis was 34 weeks. In accordance with the ACOG criteria [1], 69 cases (69%) were classified as PE without severity criteria, and 31 (31%) patients showed severity criteria (see the Section 2 for details of PE classification). A total of 21 (21%) patients had severe PE according to the previous PE severity criteria, which considered urine protein levels among their parameters [53]. For the PE classification, according to the time of onset, the cohort point was 32 WG [53,54]. Early onset PE was present in 38% of cases. Maternal age and history of a previous pregnancy with PE, as well as systolic and diastolic blood pressure, were statistically different between cases and controls (*p* < 0.05).

Table 2 compares selected laboratory results by study group. There were differences in creatinine (*p* = 0.022) and platelet counts (*p* = 0.038) with higher means in the controls. For proteins (dipstick in a single urine sample and in peripheral blood), the average of proteins for the group of cases was 72 mg/dL, while that in controls was 3 mg/dL (*p* = 0.001).

### 3.2. Genotyping of the 5-HTTLPR Genetic Variant by End-Point Polymerase Chain Reaction (PCR)

To test deviations of HW law, and prior to the assessment of the genetic association analyses, the genotype frequencies for the interest variant were obtained for 100 non-related donors (see the Materials and Methods section for details). The genotype frequencies were 40%, 26.8%, and 9.75% for the S/S, S/L, and L/L genotypes, respectively. Results showed that the study population was found to be in HW equilibrium (X^2^ = 0.806 and *p* > 0.05).

To evaluate the association between the 5-HTTLPR gene variant and PE, co-dominant, dominant, and recessive models of inheritance were used. The findings are described below, and the model of inheritance to which they correspond is also specified. Frequencies for the L/L, L/S, and S/S genotypes were 15%, 53%, and 32% for the cases and 20%, 55%, and 25% in the controls, respectively (Table 3). When comparing the frequencies of the S/S vs. S/L genotype between the study groups using the co-dominant inheritance model, a significant difference was found (*p* ≤ 0.001). Additionally, a significant difference was found when comparing the frequencies of the S/L vs. L/L genotype (*p* = 0.021). There were no differences in the proportion of S/S and L/L genotypes between the study groups (*p* = 0.67).

The distribution of the minor allele frequencies (L) was 41.5% and 32.5% for PE cases and controls, respectively, with no significant differences between the groups (*p* = 0.08) (Table 3).

Among cases with PE with severity criteria, the frequency of L/L genotype was 19.3%; for S/L, it was 48.4%; and for S/S, it was 32.3%; the corresponding frequency for cases without severity criteria was 13%, 55.1%, and 31.9%, respectively. Regarding the time of onset of PE, the frequencies of the L/L, S/L, and S/S genotypes in the early-onset PE group were 18.42%, 44.74%, and 36.84%, and in the late-onset PE group, they were 12.9%, 58.1%, and 29.0%, respectively (Table 4).

In order to evaluate the behavior of proteinuria, patients were stratified as mild PE and severe PE according to the previous ACOG classification [1]. Only 39 participants had protein data. A total of 18 (46.2%) patients had mild PE and 21 (53.8%) severe PE. Of the patients with mild PE, none presented the L/L genotype, while 8 (44.4%) were S/L and 10 (55.6%) were S/S. Of the patients with severe PE, 2 (9.5%) presented the L/L genotype, 13 (61.9%) the S/L, and 6 (28.6%) S/S. Table 4 displays the results of the comparisons of genotype frequencies between study groups using the recessive inheritance model. When comparing genotype frequencies using the recessive model of inheritance (S/S vs. S/L + L/L) between cases and controls, the S/S genotype was found to reduce the probability of developing PE (OR = 0.39, 95% CI: 0.22–0.69, *p* = 0.002). The same was found for cases without severity criteria (OR = 0.38, 95% CI: 0.21–0.73, *p* = 0.005), with severity criteria (OR = 0.39, 95% CI: 0.17–0.91, *p* = 0.045), with severe PE (OR = 0.33, 95% CI: 0.12–0.91, *p* = 0.05), and with late-onset PE (OR = 0.34, 95% CI: 0.17–0.66, *p* = 0.002) (Table 4).

Finally, a comparison of genotype frequencies between different PE sub-classifications was carried out, but no significant differences for any of the comparisons were observed (Supplementary Table S1).

## 4. Discussion

Considering the multiple effects of 5-HT on the cardiovascular, immune, gastrointestinal, central nervous, metabolic, and vascular systems and the fact that genetic variants in SERT influence the bioavailability of 5-HT in the extracellular space, the objective of this study was to evaluate the association between the 5-HTTLPR variant of the SLC6A4 gene and the occurrence of PE.

In agreement with other studies, personal history of PE was the most important risk factor associated with PE; however, if maternal age showed differences between groups, the maternal age range in both PE cases and controls was less than 35 years old, which is considered as the cut-off for increased risk for PE development in several studies [57,58,59]. Regarding the biochemical findings, in our study, the mean creatinine values between the PE cases and controls had statistical differences (0.58 mg/dL vs. 0.64 mg/dL; *p* = 0.022). However, this result is not unexpected because both groups of values were in the range of normal concentrations since creatinine during normal evolutionary pregnancy ranges from 0.5 to 0.8 mg/dL. In the same way, most of the patients with PE in our study did not present severity criteria 69 (69%), and only 38 (38%) developed early PE, which is generally associated with greater developmental impairment and worse maternal outcomes [39,40]. It has been reported that a value > 1.1 mg/dL is the cut-off point to consider renal involvement and take it into account as a diagnostic criterion when there is no proteinuria [37]. Our results agree with that reported by Vázquez et al. in 2018 [60], who evaluated 100 patients with PECS where the overall mean creatinine was 0.25 ± 0.77 mg/dL (*p* < 0.05) and only 14% reached creatinine concentrations ≥ 1.1 mg/dL [60].

In our study, the genotyping results of the 5-HTTLPR variant showed that the S/S genotype of SERT decreased the probability of PE 0.39-fold, and such a finding persisted among similar patients with PE with severity criteria (OR = 0.39, 95% CI = 0.17–0.91), and patients with severe PE (0.33-fold). These results were unexpected; however, they seem to indicate that the S/S genotype not only has an effect at the level of the uterine vessels causing vasoconstriction, as mentioned by Bolte et al. in 2001 [41], but its action could be starting in the previous stages of pregnancy. For instance, in the first trimester, the syncytiotrophoblastic layer provides a continuous barrier to passive diffusion towards the chorionic villi, through which a continuous diffusion of 5-HT is generated [61,62,63]. The excessive release then causes a turbulent flow with increased endothelial destruction of the uterine vessels, contributing with endovascular trophoblasts to the production of apoptosis in endothelial cells, allowing their arrival to the arterial smooth muscle cells [64]. This could improve the depth of trophoblast implantation, which will, in turn, increase uteroplacental blood flow [65]. In 2016, Thompson et al. reported that lymphoblastic cells with L/L genotype present much higher levels of 5-HT uptake compared with S/S or S/L cells [48], leaving more 5-HT free to act as an immunomodulator [66]. In addition, 5-HT, 5-HTT, and molecules or enzymes of the 5-HT synthesis cascade are expressed in many types of immune cells, including dendritic cells, mast cells, T-cells, B-cells, neutrophils, macrophages, monocytes, and NK cells [21]. In fact, NK cells are the most important cells at the time during trophoblast invasion [64]; therefore, when 5-HT interacts with uterine NK cells, it could modulate better implantation. However, since PE has a multifactorial etiology, the amount of free 5-HT alone would unlikely explain the pathology [64].

In a study conducted in Chile, Carrasco et al. reported that plasma 5-HT concentration was higher in women with PE compared with non-pregnant but found no difference in plasma levels of 5-HT between those with and without PE [67]. Although genetic associations of SERT were not assessed in that study, their findings point to the fact that regardless of the genotype presented by the mother, plasma 5-HT levels did not show a significant value (*p* < 0.05) in patients with established PE. In another study carried out by Sabolovic et al. in Croatia, platelet 5-HT concentrations were analyzed in pregnant women with PE, in patients with gestational hypertension without proteinuria, and in healthy pregnancies without complications (i.e., controls) [45]; their findings showed that compared with controls, there was a low platelet 5-HT level in PE- and pregnancy-induced hypertension, but unlike us, they did not find differences in the proportions of genotypes of 5-HTTLPR (tri-allelic version) between study groups. Actually, we are not aware of any study evaluating the association between the 5-HTTLPR variant of the SLC6A4 gene and PE and reporting that the S/S genotype decreases the probability of PE. Our findings also contrast with those of Sabolovic et al., as they found no difference in genotype frequencies between controls and women with PE [45]. Differences observed across studies may be due to the use of different PE definitions/classifications, the number of controls per case, the genotyping of SERT used, and the methodology and statistical analyses. For instance, most studies evaluating 5-HTTLPR variants take the S/S genotype as the risk genotype and the L/L genotype as the wild-type genotype in the statistical analysis; also, case–control studies employed to compare results use other selection criteria. For example, Sabolovic et al. included women with late PE and did not sub-classify patients with or without severity criteria [45]; Ugun-Klusek et al. evaluated placenta samples [68]; and Carrasco et al. selected only eight patients of 36 GW, with a BP > 150/100 mmHg and proteinuria (6.8 + 2.2 g in 24 h) [67]. Therefore, this seems to have influenced the modeling of data. The type of genetic inheritance model used is also relevant; in this study, the recessive model adjusted the data better, while other studies often use the co-dominant genetic inheritance model.

It is important to mention that the evaluation of IUGR using birth weight could have produced a broader range of associations than only the use of PE classifications and/or proteinuria. However, these data were not available for this study, which may be considered an important limitation. Additional studies will be necessary to evaluate the influence of the 5-HTTLPR of SLC6A4 on newborn clinical findings. On the other hand, in this study, most of the clinical information about the patients and, in some of them, the changes in systemic blood pressure were available; therefore, according to our results, it will be important to carry out a larger cohort, in order to evaluate more robustly the potential effect of the genetic variant 5-HTTLPR of the SLC6A4 gene and PE, the pregnancy outcome, and/or on clinical findings of the newborn.

The 5-HT molecular pathway is known to play a pivotal role in numerous biological mechanisms, including chronic pelvic pain in endometriosis [69]. In this context, 5-HT has been linked to a range of other neurotransmitters [70]. This observation is especially noteworthy considering that endometriosis is associated with an increased risk of placental defects [71]. Other conditions related to an altered serotoninergic pathway, such as irritable bowel syndrome and fibromyalgia [72], have also been linked to PE [73]. The mechanistic evidence for 5-HT modulation in diseases associated with PE underscores the significance of our research and indicates a direction for future studies.

Finally, it is important to mention that this study is the first that looks at the relationship between the genetic variant 5-HTTLPR of the SLC6A4 gene and PE, showing that the S/S genotype reduced the rate of PE by 0.39. Because this study is the first carried out in a Mexican population, our findings could be extrapolated to the general population, creating a new screening strategy from the genetic pathway, which is currently a well-accepted genetic theory to explain a proportion of cases of PE.

## 5. Conclusions

There was a positive association between the 5-HTTLPR gene variant of the SLC6A4 gene and PE. This relationship showed that the S/S genotype reduced the rate of PE by 0.39. This finding was similar for patients with PE with severity criteria (OR = 0.39, 95% CI: 0.17–0.91, *p* = 0.045), in patients with severe PE (OR = 0.33, 95% CI: 0.12–0.91, *p* = 0.05), and in patients with late-onset PE (OR = 0.34, 95% CI: 0.17–0.66, *p* = 0.002), respectively. Therefore, the 5-HTTLPR genetic variant of the SLC6A4 gene modifies the risk of PE among the studied population.

## Figures and Tables

**Table 1:** General characteristics of the study population (*n* = 200).

Characteristic	PE Cases (*n* = 100)	Controls (*n* = 100)	*p*-Value	OR	95% CI
Mean maternal age in years (min–max)	28 (23.0–32.0)	25.0 (23.0–29.0)	**0.026**	-	-
Weeks of gestation at diagnosis	34 (31.0–37.0)	31.0 (24.7–35.0)	**<0.001**	-	-
Primiparous, *n* (%)	47 (55.95)	37 (44.04)	0.197	1.51	0.85–2.65
Family history of PE, *n* (%)	8 (8)	9 (9)	1	-	-
Personal history of PE, *n* (%)	13 (13)	2 (2)	**0.007**	7.32	1.60–33.35
Personal history of HBP, *n* (%)	1 (1)	0 (0)	0.976	-	-
Smoking history, *n* (%)	7 (36.8)	12 (63.15)	0.335	0.55	0.20–1.46
History of alcohol use, *n* (%)	1 (1)	0 (0)	0.38	-	-
SBP in mm/Hg (min–max)	150 (140–160)	100 (100–110)	**<0.001**	-	-
DBP in mm/Hg (min–max)	90 (90–100)	70 (60–70)	**<0.001**	-	-
PE with severity criteria *n* (%)	31 (31.0)	-	-	-	-
Early onset PE *n* (%)	38 (38.0)	-	-	-	-
* Severe PE, *n* (%)	21 (53.8)	-	-	-	-

Significant *p*-values (*p* < 0.05) are highlighted in bold. * Classification according to previous ACGO criteria [53]. PE—pre-eclampsia; OR—odds ratio; HBP—high blood pressure; SBP—systolic blood pressure; DBP—diastolic blood pressure.

**Table 2:** Comparison of biochemical parameters between study groups.

Parameter	PE Cases (*n* = 100)	Controls (*n* = 100)	*p*-Value
Serum creatinine (mg/dL)	0.58 (0.48–0.7)	0.64 (0.56–0.74)	**0.022**
Plasma platelets (10^3^/μL)	214.2 (162.5–256.8)	235.0 (194–278.3)	**0.038**
Serum aspartate aminotransferase (IU/L)	29.0 (22.9–34.0)	25.0 (16.5–29.0)	0.208
Urine protein (mg/dL)	72 (46.7)	3 (39.6)	**0.001**

For creatinine, the data were available for 87 cases and 35 controls; for platelets, the data were available for 92 cases and 72 controls; for aspartate aminotransferase, data were available for 74 cases and 4 controls; for protein, data were available for 72 cases and 62 controls. *p*-values < 0.05 are highlighted in bold.

**Table 3:** Comparison of genotypic frequencies of the 5-HTTLPR variant between case and control groups using the codominant inheritance model.

Genotype/Allele	PE Cases (*n* = 100)	Control (*n* = 100)	* *p*-Value	OR	95% CI
S/S, *n* (%)	32 (32)	55 (55)	Reference	-	-
S/L, *n* (%)	53 (53)	25 (25)	**≤0.001**	0.27	0.14–0.52
L/L, *n* (%)	15 (15)	20 (20)	0.68	0.78	0.35–1.72
Allele S, *n* (%)	117 (58.5)	135 (67.5)	0.08	0.68	0.45–1.02
Allele L, *n* (%)	83 (41.5)	65 (32.5)			

* The *p*-value refers to the OR, and it was calculated considering the S/S genotype as reference; the calculation was carried out using the codominant model. *p*-values with significant differences (*p* < 0.05) are highlighted in bold. OR—odds ratio; CI—confidence interval.

**Table 4:** Comparison of genotype frequencies of the 5-HTTLPR variant between study groups using the recessive inheritance model.

Group	*n*	Genotype *n* (%)		* *p*-Value	OR	95% CI
		S/S	S/L + L/L			
Control	100	55 (55)	45 (45)	Reference	-	-
PE	100	32 (32)	68 (68)	**0.002**	0.39	0.22–0.69
PE without severity criteria	69	22 (31.9)	47 (68.1)	**0.005**	0.38	0.21–0.73
PE with severity criteria	31	10 (32.3)	21 (67.7)	**0.045**	0.39	0.17–0.91
Mild PE- based on proteinuria	18	10 (55.6)	8 (44.4)	0.83	1.02	0.37–2.80
Severe PE- based on proteinuria	21	6 (28.6)	15 (71.4)	**0.05**	0.33	0.12–0.91
Early PE	38	14 (36.8)	24 (63.3)	0.086	0.48	0.22–1.03
Late PE	62	18 (29)	44 (71)	**0.002**	0.34	0.17–0.66

* The *p*-value refers to the OR and was calculated considering the control group as reference; the calculation was performed using the recessive model (S/S vs. S/L + L/L genotype ratio). *p*-values with significant differences (*p* < 0.05) are highlighted in bold.

## Data Availability

The data presented in this study are available on request from the corresponding author.

## Supplementary Materials

The following supporting information can be downloaded at https://www.mdpi.com/article/10.3390/jpm13111535/s1, Table S1: Comparison of genotype frequencies of the 5-HTTLPR variant between subgroups of PE cases using the recessive inheritance model.
