# Supplementary material for: The S/S Genotype of the 5-HTTLPR (Serotonin-Transporter-Linked Promoter Region) Variant of the SLC6A4 Gene Decreases the Risk of Pre-Eclampsia
Source: J Pers Med. 2023 Oct 26;13(11):1535. doi: 10.3390/jpm13111535 (PMC10671924; doi:10.3390/jpm13111535)
Supplement: Supplementary file 1 [file jpm-13-01535-s001.zip › jpm-2565324-supplementary.pdf]

SUPPLEMENTARY MATERIAL -

Supplementary Table S1. Comparison of genotype frequencies of the 5-HTTLPR variant between subgroups of PE cases using the recessive inheritance model.

Group	n	Genotype n (%)		*P-value	OR	95% CI
		S/S	S/L + L/L			
PE without severity criteria	69	22 (31.9)	47 (68.1)	0.85	1.02	0.41 – 2.52
PE with severity criteria	31	10 (32.3)	21 (67.7)			
Mild PE -based on proteinuria	18	10 (55.6)	8 (44.4)	0.17	0.32	0.09 – 1.21
Severe PE-based on proteinuria	21	6 (28.6)	15 (71.4)			
Early PE	38	14 (36.8)	24 (63.3)	0.55	1.43	0.61 – 3.36
Late PE	62	18 (29)	44 (71)			

*P-value refers to OR, which comes from calculating it by comparing the proportions of the genotype frequencies between each pair of groups using the recessive model (S/S vs. S/L+L/L).
